# The effect of shunt surgery on corticospinal excitability in idiopathic normal pressure hydrocephalus: a transcranial magnetic stimulation study

**DOI:** 10.1186/s12987-022-00385-1

**Published:** 2022-11-08

**Authors:** Jani Sirkka, Laura Säisänen, Petro Julkunen, Mervi Könönen, Elisa Kallioniemi, Ville Leinonen, Nils Danner

**Affiliations:** 1 Neurosurgery, Institute of Clinical Medicine, University of Eastern Finland, Kuopio, Finland; 2 Neurosurgery, Neurocenter, Kuopio University Hospital, Kuopio, Finland; 3 Department of Clinical Neurophysiology, Kuopio University Hospital, Kuopio, Finland; 4 Department of Applied Physics, University of Eastern Finland, Kuopio, Finland; 5 Department of Clinical Radiology, Kuopio University Hospital, Kuopio, Finland; 6 Department of Psychiatry, University of Texas Southwestern Medical Center, Dallas, TX USA

**Keywords:** Idiopathic normal pressure hydrocephalus, Navigated transcranial magnetic stimulation, Corticospinal excitability, Shunt surgery, Surgical outcome

## Abstract

**Background:**

Idiopathic normal pressure hydrocephalus (iNPH) is a multifactorial disease presenting with a classical symptom triad of cognitive decline, gait disturbance and urinary incontinence. The symptoms can be alleviated with shunt surgery but the etiology of the symptoms remains unclear. Navigated transcranial magnetic stimulation (nTMS) was applied to characterize corticospinal excitability and cortical motor function before and after shunt surgery in order to elucidate the pathophysiology of iNPH. We also aimed to determine, whether nTMS could be applied as a predictive tool in the pre-surgical work-up of iNPH.

**Methods:**

24 patients with possible or probable iNPH were evaluated at baseline, after cerebrospinal fluid drainage test (TAP test) and three months after shunt surgery (follow-up). Symptom severity was evaluated on an iNPH scale and with clinical tests (walking test, Box & Block test, grooved pegboard). In the nTMS experiments, resting motor threshold (RMT), silent period (SP), input–output curve (IO-curve), repetition suppression (RS) and mapping of cortical representation areas of hand and foot muscles were assessed.

**Results:**

After shunt surgery, all patients showed improved performance in gait and upper limb function. The nTMS parameters showed an increase in the RMTs (hand and foot) and the maximum value of the IO-curve increased in subject with a good surgical outcome. The improvement in gait correlated with an increase in the maximum value of the IO-curve. SP, RS and mapping remained unchanged.

**Conclusion:**

The excitability of the motor cortex and the corticospinal tract increased in iNPH patients after shunt surgery. A favorable clinical outcome of shunt surgery is associated with a higher ability to re-form and maintain neuronal connectivity.

**Supplementary Information:**

The online version contains supplementary material available at 10.1186/s12987-022-00385-1.

## Background

Idiopathic normal pressure hydrocephalus (iNPH) is a progressive multifactorial disease of the elderly population [1]. The prevalence of iNPH has been suggested to be as high as 5.9% in people aged 80 years or older [2]. INPH can be recognized from its classical symptom triad, which includes disturbances in urinary continence, cognition and gait. Typically, impairment in gait is the initial and the most prominent symptom [1]. Recently, also upper limb motor dysfunction has been strongly associated with the symptomatology of iNPH [3].

INPH can be treated with surgery in which an artificial pathway for cerebrospinal fluid (CSF) is created [4, 5]. However, one of the greatest challenges of the treatment is to choose those patients who will benefit from surgery [6]. Previous studies have shown that the likelihood of a positive surgical outcome varies from 28 to 100% [7] depending on patient selection and criteria for shunting. Accordingly, patient selection is a matter of balance between possible surgical complications and potential benefit [8]. The outcome of surgery may be predicted with invasive preoperative tests, such as a cerebrospinal fluid drainage test (TAP test), in which CSF is drained to simulate a shunt effect, or a lumbar infusion test, both of which have only a moderate accuracy [9].

The precise neuronal background of iNPH and the mechanisms by which shunt surgery alleviates the symptoms are still unclear, but the literature suggests that iNPH might affect corticospinal excitability, which can be altered by drainage of CSF or permanently with shunt surgery. Studies have suggested that the symptoms are related to intracortical inhibition and synaptic connectivity of the motor cortex [10, 11]. In this study, we aim to characterize the effects of shunt surgery on corticospinal excitability with navigated transcranial magnetic stimulation (nTMS). TMS is a non-invasive brain stimulation method, which activates cortical motoneurons and corticospinal pathways. Different aspects of cortical excitation and inhibition may be addressed by applying specific stimulation protocols [12]. Neuronavigation combined with TMS allows corticospinal excitability to be followed up in repeated examinations even if anatomical changes occur due to the underlying pathology or treatment [13]. Therefore, nTMS is ideally suited for studying iNPH, in which the anatomy of the cerebral ventricles and the cortex is altered by the pathology and further influenced by shunt surgery [14, 15].

In addition to studying treatment-related changes in motor cortical function and corticospinal excitability we also aimed to determine, whether nTMS could be applied as a predictive tool in the pre-surgical work-up of iNPH.

## Methods

### Study population

**Fig. 1:**
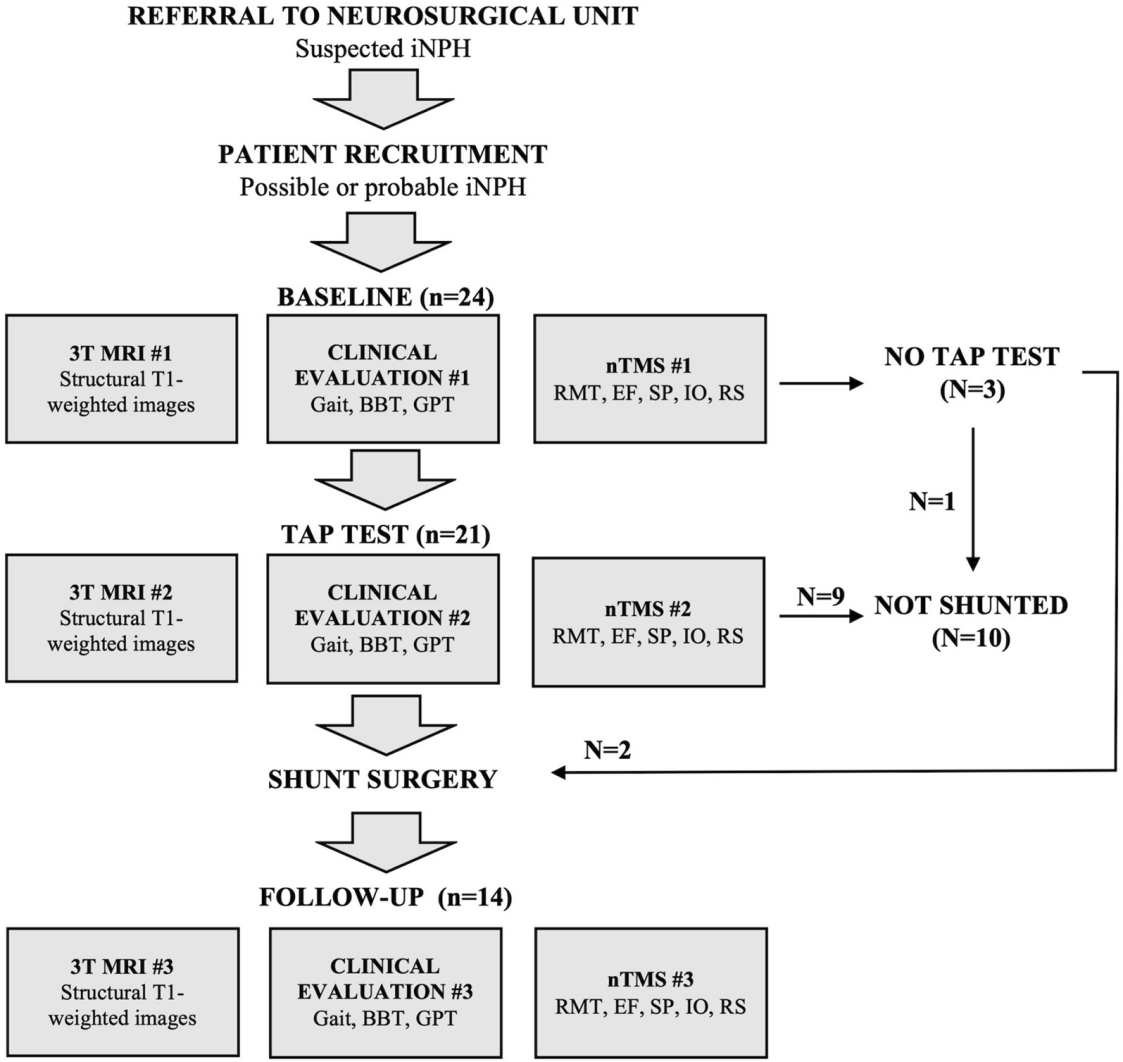
Flow chart of the study. 3 T *MRI* 3 Tesla magnetic resonance imaging, *iNPH* idiopathic normal pressure hydrocephalus, *nTMS* navigated transcranial magnetic stimulation, *SP* silent period, *RMT* resting motor threshold, *EF* electric field, *IO* input–output curve, *RS* repetition suppression, *BBT* box and block test, *GPT* grooved pegboard test

Twenty-four patients were recruited from an ongoing prospective iNPH study at Kuopio University Hospital (KUH). The patients had previously undergone neurological evaluation and were referred to KUH for neurosurgical evaluation due to suspected iNPH (Fig. 1). All patients met the criteria of probable or possible iNPH in accordance with the classic symptom triad and brain imaging [1]. Neurological comorbidities [1], medications with effects on TMS responses [16] and Mini-Mental State Examination (MMSE) [17] score under 20 points were regarded as exclusion criteria of this study [10]. In the study population, a disproportionately enlarged subarachnoid space (DESH) was radiologically detected in three patients [18]. The patients had following non-neurological co-morbidities: hypertension (n = 16), hypercholesterolemia (n = 13), coronary artery disease (n = 9), adult-onset diabetes (n = 6), osteoporosis (n = 4), hypothyroidism (n = 4), arthrosis (n = 3), vitamin B12 deficiency (n = 3), atrial fibrillation (n = 2), depression (n = 2), asthma (n = 2), prostate hyperplasia (n = 2), psoriasis (n = 1), rheumatoid arthritis (n = 1) and fibromyalgia (n = 1).

### Clinical assessments

Clinical assessments included an evaluation of gait, upper limb motor function and iNPH scale [19]. Gait was evaluated as an average time of three trials in a 10-m walking test. Upper limb fine motor performance was evaluated by the used time in the Grooved Pegboard test (GPT) in which key shaped pegs are placed in holes with randomly positioned slots. Gross motor function of upper limb was assessed with the Box And Block test (BBT) in which cubes (2.5 cm side) are moved from one box to another within a time-limit of 60 s [20]. The gait test and GPT are a part of iNPH scale which also includes evaluation of balance, continence and cognition. The total score on the iNPH scale (max 100) was calculated and weighted between tests in accordance with previously published guidelines [19].

### Radiological imaging

Magnetic resonance imaging (MRI) was performed with a 3T MRI scanner (Philips Achieva, The Netherlands). Structural three-dimensional T1-weighted images with 1 mm^3^ voxel size were used for TMS navigation. The distance from scalp to cortex was manually determined from MR-images. The measurement was made from the outermost part of the “hand knob” when determining the distance to hand representation area [21, 22]. For the representation are of the foot, the distance from the scalp was measured to the outermost part of the precentral gyrus adjacent to the midline.

### Navigated transcranial magnetic stimulation

NTMS was performed with an NBS system (version 4.3.1, Nexstim Plc, Helsinki, Finland) using a figure-of-eight coil with biphasic pulse waveform. The stimuli were targeted to the primary motor cortex of the left hemisphere. TMS-induced motor evoked potentials (MEPs) were recorded from contralateral peripheral muscles by integrated stimulus-locked electromyography (EMG). The targeted muscles were the first dorsal interosseus (FDI) when stimulating the representation area of the hand and the abductor hallucis brevis (AHB) when stimulating the representation area of the foot. Other muscles (abductor pollicis brevis, extensor carpi radialis, tibialis anterior and vastus lateralis) were monitored with EMG to minimize simultaneous muscle activation. The approval limit of MEPs was 50 µV (peak-to-peak amplitude) and all responses with preceding or concurrent muscle activation 50 ms before the stimulus were excluded from the analysis [23].

The nTMS protocol was executed in accordance with International Federation of Clinical Neurophysiology guidelines [12]. The hotspots for FDI and AHB, producing the MEPs with the highest amplitudes, were mapped from the primary motor cortex as previously described [10]. An integrated aiming tool was used to rotate the coil at the hotspot in order to determine the optimal orientation. The cortical representation areas for FDI and AHB were mapped starting from the hotspots with the aid of a virtual grid (5 mm × 5 mm per square) and a stimulator intensity 105% of RMT [24]. Two stimulations with approximately 5 s interval were applied to each square of the grid until no MEPs were induced at the outer borders of the formed area. The representation areas were calculated using the spline interpolation method [24, 25]. The centers-of-gravity (CoGs) [26] for motor representations were determined using the MRI coordinate space in the navigation system, which has the origin in the right-posterior-inferior corner of the image. Corticospinal excitability was evaluated by determining the resting motor threshold (RMT) using an integrated iterative threshold assessment tool and reported as stimulator output percentage (% of maximum stimulator output, %-MSO) as well as calculated electric field (EF) strength (V/m) [13, 27, 28]. Corticospinal inhibition was evaluated by the silent period (SP), which is a stimulation induced break in voluntary muscle activation [29]. SPs were elicited at 120% of the RMT [30] and determined as an average of five trials. The Input–Output (IO) curve was determined from a sequence of 160 pulses (intensities from 80 to 150% of RMT in 10% steps and intervals from 3 to 5 s) in a randomized order. A Boltzmann sigmoidal function was used to determine the maximum value (IO-max), the mid-point of the curve (V50) and the slope of the curve (IO-slope) [10, 31, 32]. The ability of the cortex to habituate to repetitive stimuli was assessed by repetition suppression (RS) paradigm, in which 120 stimuli were delivered in trains of four stimuli at 120% of RMT with stimulation interval of 1 s and trains repeated every 20 s. [33]. The four MEP amplitudes within the RS trials were averaged over all trials to represent RS response with second to fourth MEP in the trials representing the suppressed level of RS [34].

### Flow of the study

The study protocol consisted of three identically repeated measurement sessions including clinical testing, MRI-imaging and TMS evaluation at three different time points (Fig. 1). In the first session of this study, baseline measurements were made at the neurosurgical outpatient clinic (baseline). On the following day a TAP test was conducted by draining 40 ml of CSF via lumbar puncture [9, 35]. The second measurement session was conducted one to two hours after the drainage (TAP) [36]. The third session was conducted three months after shunt surgery (follow-up). The decision for surgical treatment was done in accordance with the KUH iNPH protocol [36]. The clinical protocol includes an assessment of symptoms, cognitive testing, MRI and a TAP test. An improvement of 20% in walking speed is considered as a positive TAP test result but is not regarded as a cut-off limit for shunt surgery. Adjustable and MRI-compatible shunt valves (Miethke proGAV 2.0, Christoph Miethke GmbH & Co., Potsdam, Germany) with a setting of 25 cmH_2_O vertical and 5 cm H_2_O horizontal were used for all patients.

### Analysis and statistics

The differences between repeated measurements of all parameters at different time points were compared with the Wilcoxon signed-rank test, since most of the parameters were non-normally distributed (according to the Kolmogorov–Smirnov and Shapiro–Wilk tests). Changes in each parameter from baseline to TAP and from baseline to follow-up were calculated and Spearman’s test was used to assess correlations between these changes. Clinical and TMS parameters at each time-point were compared between the subgroups with the Mann–Whitney U test. P-values < 0.05 were considered statistically significant. Statistical analyses were performed with SPSS (Version 27.0; IBM Corporation, Somers NY).

## Results

### Baseline and selection for shunt surgery

Of the 24 recruited patients 21 underwent a TAP test. Ten patients were clinically evaluated not to benefit from surgery based on pre-surgical testing [36]. Eventually, 14 patients were shunted (3 men and 11 women, mean age 74.6 ± 4.2 years, range 69–84 years). One patient required revision due to shunt failure and follow-up examinations were made three months after the shunt revision. The shunted patients had more severe symptoms reflected as slower performance in the walking test at baseline (*p* = 0.046) and in the TAP test (*p* = 0.049) as compared to the non-shunted patients. In the TMS parameters, only the suppressed level MEP amplitudes in RS differed between the shunted and non-shunted patients at baseline (2nd MEP: *p* = 0.049 and 3rd MEP: *p* = 0.030). At baseline, BBT correlated with SP (*rho* = 0.558, *p* = 0.006) in all patients. There were no other correlations between clinical parameters and TMS parameter at baseline. Detailed values of the parameters for shunted and non-shunted patients are presented in Additional file 1.

### TAP test and surgical outcome at follow-up

**Fig. 2:**
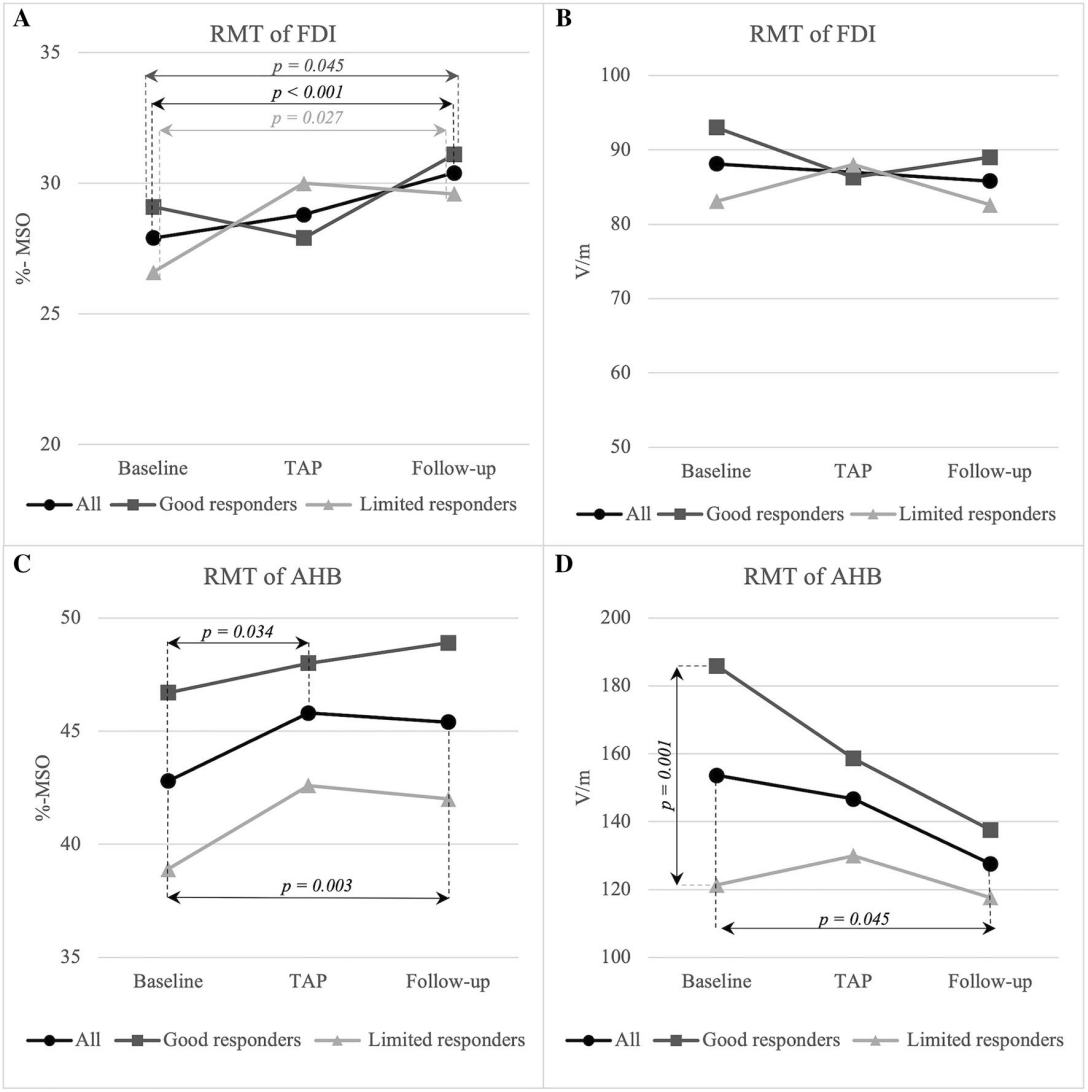
Resting motor threshold of shunted patients at baseline, TAP and follow-up. *RMT *resting motor threshold, *FDI* first dorsal interosseus, *AHB* abductor hallucis brevis, *%-MSO* percentage of maximum stimulator output, *TAP* lumbar tap test, *V/m* volt per meter

**Fig. 3:**
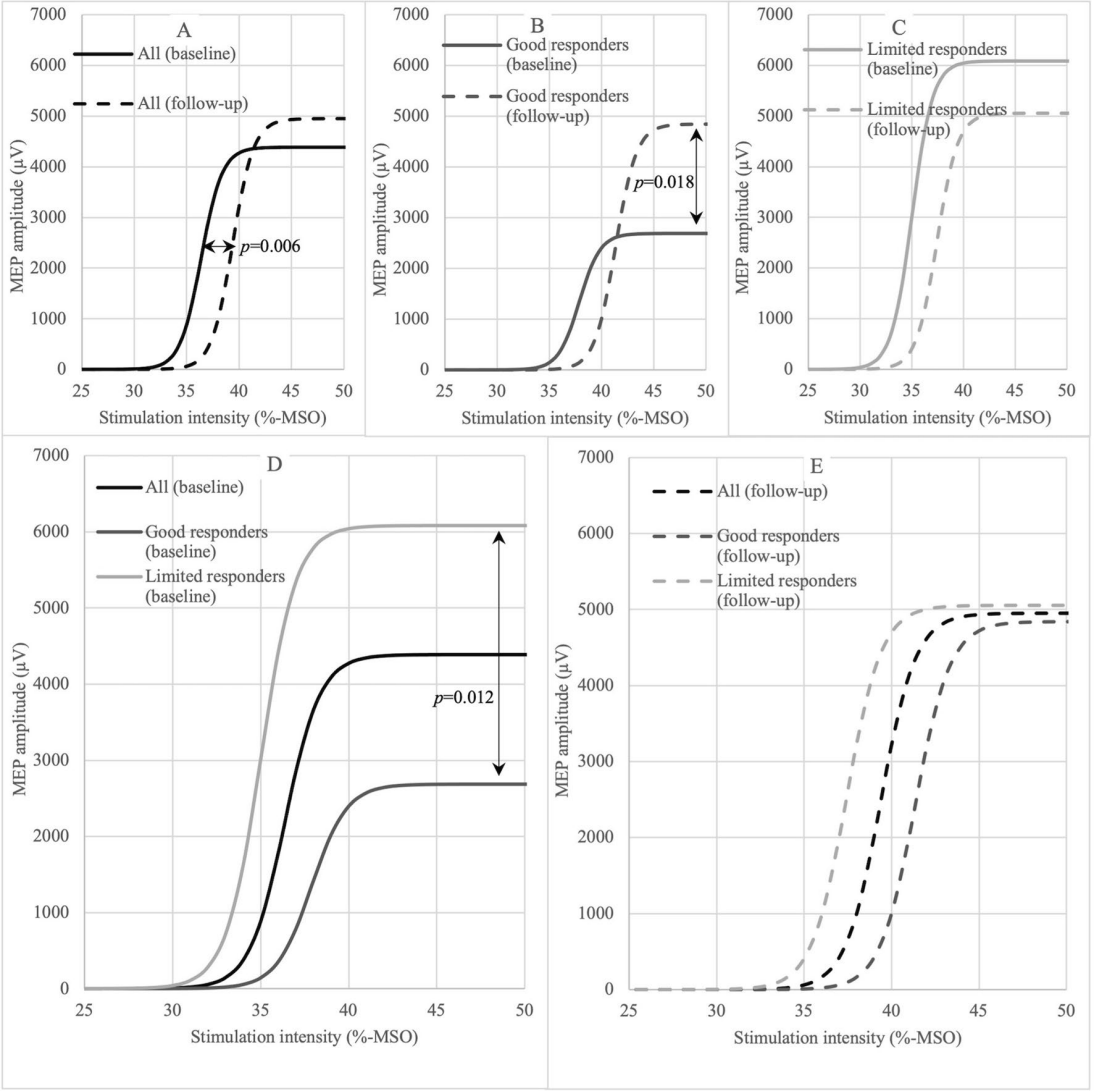
Input–Output curves of shunted patients. All patients (**A**), good responders (**B**) and limited responders (**C**) at baseline (**D**) and at follow-up (**E**). A Boltzmann sigmoidal function was used to calculate the Input–Output curves. MEP = Motor evoked potential, %-MSO = percentage of maximum stimulator output V50 (the mid-point of the curve) increased in all patients from baseline to follow-up (*p* = 0.006), at baseline IO-max (maximum value of the curve) was lower in good responders than limited responders (*p* = 0.012) and IO-max increased in good responders from baseline to follow-up (*p* = 0.018)

The shunted patients exhibited a significant difference in walking speed after the TAP test (p = 0.012) as compared to the baseline. Lumbar puncture failed in three patients and therefore the TAP test could not be performed in them. At follow-up, the shunted patients showed significant improvement in walking time (*p* = 0.019), BBT (*p* = 0.007) and iNPH scale (*p* = 0.004). From the TMS parameters, RMT (%-MSO) of the AHB increased from baseline to TAP in the shunted patients (*p* = 0.034), whereas the EF did not change (Fig. 2C and D). From baseline to follow-up RMTs of FDI (*p* < 0.001) and AHB (*p* = 0.003) in terms of %-MSO increased whereas RMT expressed as EF of AHB decreased (*p* = 0.045) (Fig. 2A, C and D). The V50 value of the IO curve increased from baseline to follow-up (*p* = 0.006) (Fig. 3A). In addition, the COG of the AHB moved laterally (*p* = 0.002), and the scalp to cortex distance was longer after shunt surgery (FDI: *p* = 0.032 and AHB: *p* = 0.001). The change from baseline to TAP correlated with the change from baseline to follow-up (*p* < 0.05) in walking (*rho* = 0.78), BBT (*rho* = 0.67) and RMTs expressed as EF (FDI: *rho* = 0.67 and AHB: *rho* = 0.66). The improvement in walking time from baseline to follow-up correlated with the concurrent increase in IO-max (*rho* = -0.684, *p* = 0.007). Detailed values of test parameters of shunted and non-shunted patients are presented in Additional file 1 and individual data of subjects in Additional file 2.

### Good responders and limited responders

For subgroup analyzes the study population was divided into two equal sized subgroups based on the improved walking time after shunt surgery. The resulting cut-off point was 17% improvement in walking time from baseline to follow-up. The patients were divided into good responders > 17% and limited responders < 17%. Good responders showed improvement in walking from baseline to TAP (*p* = 0.018) and the improvement was also detected in walking (*p* = 0.018), BBT (*p* = 0.042) and iNPH scale (*p* = 0.018) from baseline to follow-up in contrast to limited responders. In both groups the scalp-to-cortex distance of AHB (good responders: *p* = 0.018 and limited responders: *p* = 0.018) and RMT (%-MSO) of FDI increased (good responders: *p* = 0.045 and limited responders: *p* = 0.027) (Fig. 2A) from baseline to follow-up. In good responders, IO-max increased from baseline to follow up (*p* = 0.018) (Fig. 3B). Furthermore, in good responders the change from baseline to TAP correlated with the change from baseline to follow-up (*p* < 0.05) in walking (*rho* = 0.93), BBT (*rho* = 0.85) and RMT of FDI (%-MSO: *rho* = 0.80 and EF: *rho* = 0.86). In a comparison between subgroups good responders showed significantly higher AHB RMTs (EF) (*p* = 0.001) (Fig. 2D), whereas maximum values of IO were lower than in limited responders at baseline (*p* = 0.012) (Fig. 3D). The COGs of FDI (*p* = 0.047) and AHB (*p* = 0.011) were more posteriorly located in good responders than in limited responders at baseline. Detailed values of the parameters in good and limited responders are presented in Additional file 3.

## Discussion

We characterized the effects of shunt surgery on the excitability of corticospinal pathways and evaluated the association between corticospinal excitability and motor functions in iNPH patients. After shunt surgery, patients showed improvement in walking speed and upper limb gross-motor function. The surgical outcome correlated with the response in the TAP test. The clinical response to shunt surgery correlated with an increased excitability of the motor cortex and the corticospinal pathways reflected in the TMS parameters.

### Motor thresholds

Previously, iNPH patients have been shown to present with corticospinal hyperexcitability and decreased short interval intracortical inhibition, which are linked to deficits in motor performance. These findings have been suggested to derive from impairments in neurotransmitter circuits of GABA, glutamate and choline [10, 11, 37]. On the other hand, neuronal conductivity of the corticospinal tract seems to function normally [11, 38]. In the current study, in line with previous findings [11], RMTs increased after shunt surgery indicating normalization of corticospinal hyperexcitability. However, it has been unclear whether the observed RMT increase is due to anatomical or physiological changes, or a combination of both. Shunt surgery alters the anatomy of the cortex and the ventricles and leads to an increase in the distance from the scalp to the cortex as observed in this study. According to electromagnetic principles, this increase in distance leads to a decline in the TMS-induced electric field on the stimulated cortex, which in turn results in a need for higher stimulator output in order to activate the cortex and is reflected as higher RMTs [39]. In order to eliminate the effect of anatomical changes, we also calculated the strength of the stimulus-induced EF at the cortex when determining RMT. Interestingly, shunt surgery decreased the strength of the cortical EF required to induce activation of the corticospinal tracts, which can be interpreted as an increase in cortical excitability. Even at baseline iNPH patients have been shown to require lower EFs for cortical activation than healthy control subjects [10]. In iNPH patients, who clinically present with slow gait, the observed cortical hyper-excitability is rather paradoxical and therefore likely presents a secondary process in the pathophysiology. It may demonstrate a reactive mechanism of the motor cortex to an underlying subcortical pathology, which is further enhanced with shunt surgery. In line with the surgical outcome, an initial EF decrease after the TAP test seems to be associated with a further decrease of EF at follow-up after shunt surgery.

### Input–output curve

A dose–response analysis of TMS was evaluated with the IO-curve. Across all patients, we observed an increase in the V50 value, representing the stimulator intensity to produce average-sized MEPs [31] from baseline to follow-up. The increase of V50 is indicating regression of the corticospinal hyperexcitability [40]. We also noticed that good responders elicited MEPs with a lower maximum amplitude (IO-max) at baseline, but it increased significantly with shunt surgery. In our previous study, we hypothesized that patients with a positive TAP test response could have a higher cortical recovery potential due to better preserved synaptic connectivity and plasticity [10]. The maximum amplitude of the IO-curve has been suggested to reflect the maximum capacity of recruitable motoneurons for a TMS-induced motor response [12]. In children whose brain is still developing, the IO-max is lower as compared to adolescents and adults [41] which is probably related to synaptic spinal motoneuron density [42]. From this perspective, iNPH might cause a reversible regression of corticospinal synaptic connectivity. The present study also showed that increase in IO-max due to shunt surgery is associated with the recovery of walking speed in iNPH. These patients respond well to shunt surgery and may even be recognized at baseline from the lower IO-max.

### Cortical representation area

With nTMS we were able to localize and determine the size of cortical representation areas of the FDI muscle and the AHB muscle. We did not find relevant changes or associations between the size of cortical representation areas and the clinical manifestations of iNPH. Only the COG of the AHB muscle moved laterally at follow-up, which is likely explained by the change in scalp to cortex distance. The stable cortical representation areas indicate that the actual functional area of the motor cortex is not altered in iNPH, but the observed changes are attributed to functional connectivity.

### Cortical inhibition

As a difference from previous reports, we did not recognize a reversible impairment in cortical inhibition mechanisms, reflected in the SPs, in patients with a good clinical outcome after shunt surgery [11]. As a difference, the previous study assessed short interval intracortical inhibition which is presumably mediated by gamma-amino-butyric acid A (GABA_A_) receptors and dopaminergic interneurons as compared to SP which is thought to be mostly mediated by GABA-B interneurons [43].

### Repetition suppression

Dispite the difference between in the suppressed level MEP responses in RS between the shunted and non-shunted patients, we did not find differences in the ability of the cortex to habituate to repetitive stimuli in patients who underwent shunt surgery.

### Limitations

The population of this study represents a relatively mild clinical picture of iNPH due to the rather complex methodology, which demanded fluent co-operation of the subjects. Moreover, not all patients were operated and even some of the shunted patients had only a modest surgical outcome that may reflect a diagnosis of “possible iNPH” instead of “probable iNPH” in those patients [1]. Furthermore, in accordance with the study protocol, the TAP test response was determined within 2 h, which excludes patients, who might have exhibited a delayed response. We recognize these limitations of the current study, and therefore, the results cannot be generalized to the entire clinical spectrum of iNPH. On the other hand, we specifically aimed to obtain a homogeneous patient population for this (pilot) study in order to characterize the effect of shunt surgery per se. Therefore, larger studies are warranted to evaluate the reproducibility of the results in patients with a more severe symptomatology and to verify, whether TMS can be applied as a prognostic tool.

## Conclusion

This is the first study to evaluate the effect of shunt surgery on cortical excitability in iNPH patients with navigated TMS. The TMS findings reflect a shunt-induced increase in excitability. However, it cannot be stated whether this is due to an increase in excitation and/or a decrease in inhibition. A favorable clinical outcome of shunt surgery is associated with the ability of the motor cortex and the corticospinal tracts to re-form and maintain neuronal connectivity. Navigated TMS offers a potentially promising clinical tool for predicting the surgical outcome in iNPH.

## Supplementary Information

**Additional file 1. **Data of shunted and not shunted patients at baseline, at TAP and at follow-up.

**Additional file 2. **Individual follow-up data of shunted and not shunted patients.

**Additional file 3. **Data of good responders and limited responders at baseline, TAP and follow-up.

## Data Availability

The datasets used and analyzed during the current study are available from the corresponding author on reasonable request.

## Abbreviations

AHB: Abductor hallucis brevis
BBT: Box & block test
CoG: Center-of-gravity
CSF: Cerebrospinal fluid
EF: Electric field
EMG: Electromyography
FDI: First dorsal interosseus
GABA: Gamma-amino-butyric acid
GPT: Grooved Pegboard test
IO-curve: Input–output curve
IO-max: Maximum value of the IO-curve
V50: The mid-point of the IO-curve
IO-slope: The slope of the IO-curve
iNPH: Idiopathic normal pressure hydrocephalus
KUH: Kuopio University Hospital
MEP: Motor evoked potential
MMSE: Mini-mental State Examination
MRI: Magnetic resonance imaging
MSO: Maximum stimulator output
nTMS: Navigated transcranial magnetic stimulation
RMT: Resting motor threshold
RS: Repetition suppression
SP: Silent period
TAP: Cerebrospinal fluid drainage test
%-MSO: Percentage of maximum stimulator output
