# Supplementary material for: The effect of shunt surgery on corticospinal excitability in idiopathic normal pressure hydrocephalus: a transcranial magnetic stimulation study
Source: Fluids Barriers CNS. 2022 Nov 8;19:89. doi: 10.1186/s12987-022-00385-1 (PMC9644524; doi:10.1186/s12987-022-00385-1)
Supplement: Supplementary file 1 — Additional file 1. Data of shunted and not shunted patients at baseline, at TAP and at follow-up. [file 12987_2022_385_MOESM1_ESM.docx]

|  | Baseline | | | TAP | | | ^1^Baseline vs. TAP  *p*-value | | Follow-up | ^1^Baseline vs. follow-up  *p*-value | ^3^Correlation between changes  (baseline – TAP vs. baseline – follow-up) |
| --- | --- | --- | --- | --- | --- | --- | --- | --- | --- | --- | --- |
|  | Shunted  (n=14) | Not shunted  (n=10) | ^2^*p*-value | Shunted  (n=12) | Not shunted  (n=9) | ^2^*p*-value | Shunted | Not shunted | Shunted  (n=14) | Shunted | Shunted |
| Walking  Time (s) | 13.7 ± 5.6 | 10.1 ± 1.9 | 0.046* | 12.2 ± 3.0 | 9.5 ± 2.1 | 0.049* | 0.012* |  | 10.8 ± 3.0 | 0.019* | 0.78* |
| GPT  Time (s) | 122.4 ± 27.5 | 174.3 ± 144.2 |  | 119.0 ± 24.0 | 136.8 ± 72.7 |  |  |  | 121.1 ± 26.8 |  |  |
| BBT  Number of boxes (pcs.) | 50.5 ± 9.4 | 56.1 ± 16.0 |  | 51.8 ± 10.1 | 56.7 ± 8.9 |  |  |  | 54.8 ± 10.3 | 0.007* | 0.67* |
| iNPH scale  Score | 54.2 ± 15.1 | 63.8 ± 12.9 |  | – | – | – | – | – | 63.4 ± 14.4 | 0.004* | – |
| Scalp to cortex distance (mm)  Hand  Foot | 12.8 ± 1.6  11.6 ± 1.6 | 12.6 ± 2.7  12.2 ± 2.9 |  | 12.8 ± 1.6  11.6 ± 1.7 | 12.4 ± 3.0  12.3 ± 2.5 |  |  |  | 13.7 ± 1.9  12.8 ± 1.5 | 0.032*  0.001* |  |
| RMT  Hand  %-MSO  EF (V/m)  Foot  %-MSO  EF (V/m) | 27.9 ± 6.4  88.1 ± 20.9  42.8 ± 7.7  153.6 ± 43.5 | 27.3 ± 4.9  78.7 ± 10.6  44.4 ± 9.7  129.4 ± 29.8 |  | 28.8 ± 5.5  87.0 ± 14.5  45.8 ± 8.1  146.7 ± 37.3 | 26.0 ± 4.8  82.0 ± 17.3  42.7 ± 9.3  126.2 ± 20.5 |  | 0.034* |  | 30.4 ± 6.5  85.8 ± 17.5  45.4 ± 8.5  127.6 ± 22.4 | <0.001*  0.003*  0.045* | 0.67*  0.66* |
| SP  Duration (ms) | 53.0 ± 15.3 | 61.6 ± 18.2 |  | 55.6 ± 18.8 | 56.6 ± 23.2 |  |  |  | 58.0 ± 17.5 |  |  |
| IO  MAX (μV)  V50 (%-MSO)  Slope | 4386.3 ± 2728.0  37.4 ± 9.8  2.8 ± 1.5 | 5117.1 ± 2662.9  36.7 ± 4.5  2.7 ± 1.3 |  | 3930.3 ± 2068.8  39.1 ± 7.7  3.4 ± 1.5 | 5901.2 ± 3387.6  36.5 ± 3.2  2.6 ± 0.9 |  |  |  | 4948.4 ± 2074.3  40.6 ± 11.2  3.4 ± 2.1 | 0.006* |  |
| RS  2nd MEP  3rd MEP  4th MEP  (MEP amplitudes were normalized by the amplitude of the 1^st^ MEP in the RS trials) | 0.79 ± 0.18  0.80 ± 0.25  0.79 ± 0.26 | 0.59 ± 0.23  0.60 ± 0.16  0.57 ± 0.14 | 0.049*  0.030* | 0.74 ± 0.18  0.70 ± 0.19  0.72 ± 0.19 | 0.70 ± 0.22  0.70 ± 0.11  0.70 ± 0.13 |  |  | 0.012* | 0.77 ± 0.25  0.86 ± 0.32  0.85 ± 0.40 |  |  |
| Map  Hand  Area  COG  X  Y  Z  Foot  Area  COG  X  Y  Z | 2.4 ± 2.1  126.6 ± 4.4  190.7 ± 5.3  87.5 ± 8.3  2.7 ± 2.0  106.7 ± 5.1  196.2 ± 7.9  75.5 ± 10.1 | 2.1 ± 1.2  128.8 ± 4.5  185.9 ± 7.1  84.2 ± 12.2  3.2 ± 1.4  104.1 ± 3.9  192.2 ± 7.2  74.5 ± 12.1 |  | 2.0 ± 1.6  128.7 ± 3.9  188.7 ± 5.1  86.8 ± 9.8  2.5 ± 1.4  107.0 ± 4.0  195.8 ± 8.8  75.0 ± 11.1 | 1.8 ± 1.6  128.3 ± 5.5  185.1 ± 8.8  83.3 ± 9.4  2.4 ± 1.2  103.8 ± 5.2  192.9 ± 7.6  74.6 ± 13.8 |  |  |  | 2.6 ± 1.3  125.7 ± 4.1  188.9 ± 6.3  88.5 ± 10.7  3.1 ± 1.5  102.2 ± 2.9  196.4 ± 7.7  78.7 ± 9.0 | 0.002* |  |

**Data of shunted and not shunted patients at baseline, at TAP and at follow-up.**

SP = Silent period, RMT = Resting motor threshold, EF = Electric field, IO = Input-Output curve, RS = Repetition Suppression, BBT = Box and Block Test, GPT = Grooved Pegboard Test, iNPH = idiopathic normal pressure hydrocephalus, %-MSO = percentage of maximum stimulator output, EF = Electric field, MAX = maximum value of the curve, V50 = The mid-point of the curve, Slope = the slope of the curve, MEP = motor evoked potential, Map = Mapping of the cortical representation areas, COG = centers-of-gravity (X, Y, Z -coordinates), TAP = Time point after TAP test

^1^Wilcoxon signed-rank test was used to analyze differences between each time points.

^2^Subgroups were compared by Mann-Whitney U test in each time point.

^3^Changes from baseline to TAP test and from baseline to follow up were calculated. Spearman’s test was used to analyze correlation.

* *p* < 0.05
