# Supplementary material for: The effect of shunt surgery on corticospinal excitability in idiopathic normal pressure hydrocephalus: a transcranial magnetic stimulation study
Source: Fluids Barriers CNS. 2022 Nov 8;19:89. doi: 10.1186/s12987-022-00385-1 (PMC9644524; doi:10.1186/s12987-022-00385-1)
Supplement: Supplementary file 2 — Additional file 2. Individual follow-up data of shunted and not shunted patients. [file 12987_2022_385_MOESM2_ESM.docx]

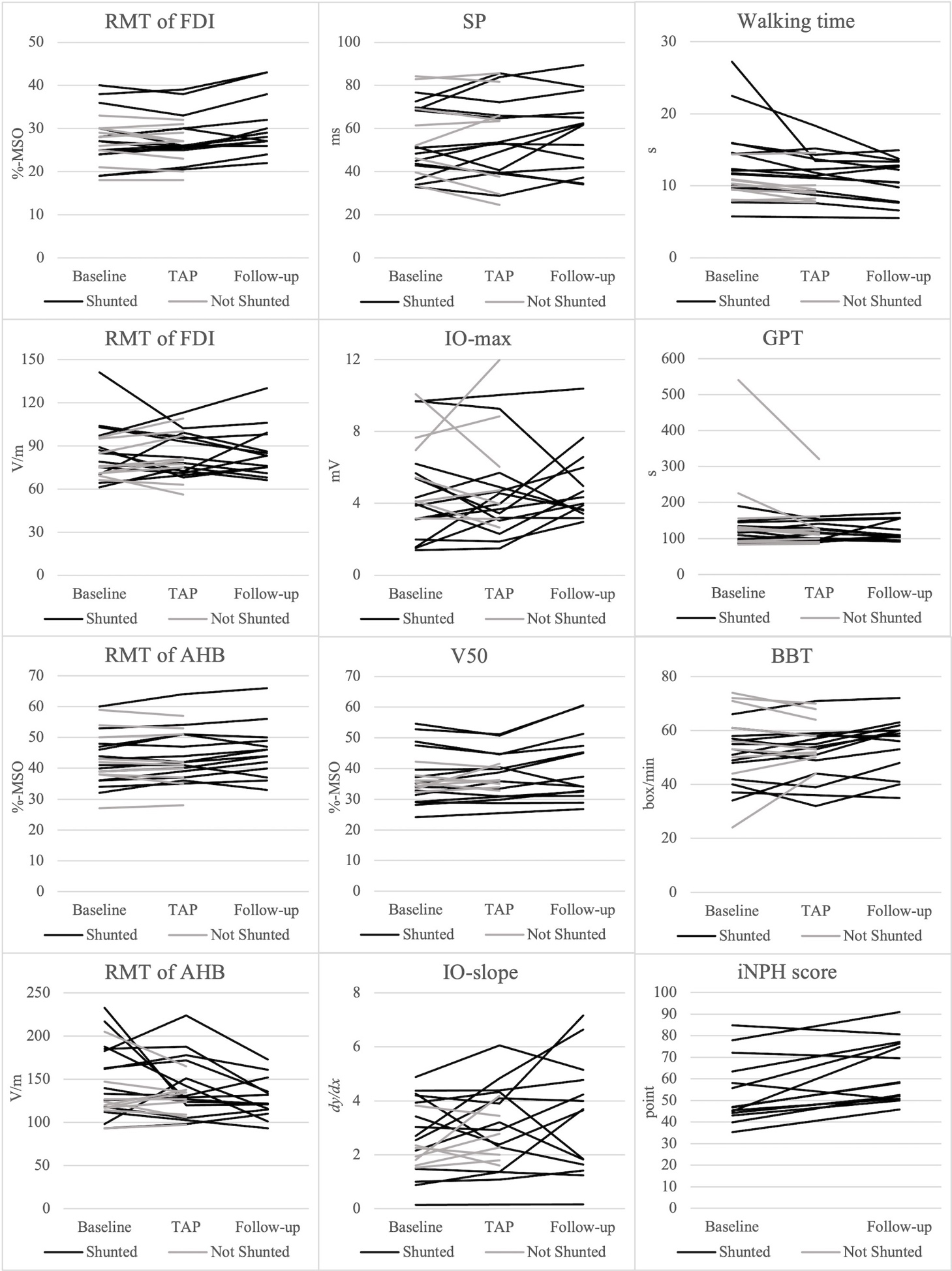


**Individual follow-up data of shunted and not shunted patients.**

SP = Silent period, RMT = Resting motor threshold, BBT = Box and Block Test, GPT = Grooved Pegboard Test, iNPH = idiopathic normal pressure hydrocephalus, %-MSO = percentage of maximum stimulator output, IO-max = maximum value of the Input-Output curve, V50 = The mid-point of the Input-Output curve, IO-slope = the slope of the Input-Output curve, FDI = first dorsal interosseus, AHB = abductor hallucis brevis, TAP = lumbar tap test
