# Supplementary material for: The effect of shunt surgery on corticospinal excitability in idiopathic normal pressure hydrocephalus: a transcranial magnetic stimulation study
Source: Fluids Barriers CNS. 2022 Nov 8;19:89. doi: 10.1186/s12987-022-00385-1 (PMC9644524; doi:10.1186/s12987-022-00385-1)
Supplement: Supplementary file 3 — Additional file 3. Data of good responders and limited responders at baseline, TAP and follow-up. [file 12987_2022_385_MOESM3_ESM.docx]

|  | Baseline | | | TAP | | | Follow-up | | | ^1^Baseline vs. TAP  *p*-value | | ^1^Baseline vs. follow-up  *p*-value | | ^2^Correlation between changes (baseline – TAP vs. baseline – follow-up) | |
| --- | --- | --- | --- | --- | --- | --- | --- | --- | --- | --- | --- | --- | --- | --- | --- |
|  | Good responders (n=7) | Limited responders (n=7) | ^3^*p*-value | Good responders (n=7) | Limited responders (n=5) | ^3^*p*-value | Good responders (n=7) | Limited responders (n=7) | ^3^*p*-value | Good responders (n=7) | Limited responders (n=7) | Good responders (n=7) | Limited responders  (n=7) | Good responders (n=7) | Limited responders (n=7) |
| Walking  Time (s) | 15.4 ± 7.2 | 12.0 ± 3.2 |  | 11.8 ± 3.7 | 12.8 ± 1.8 |  | 10.1 ± 3.0 | 11.5 ± 3.1 |  | 0.018* |  | 0.018* |  | 0.93* |  |
| GPT  Time (s) | 122.4 ± 16.9 | 122.3 ± 36.8 |  | 123.0 ± 23.6 | 113.4 ± 26.3 |  | 125.9 ± 26.6 | 116.4 ± 28.3 |  |  |  |  |  |  |  |
| BBT  Number of boxes (pcs.) | 50.9 ± 8.7 | 50.1 ± 10.8 |  | 51.0 ± 12.7 | 53.0 ± 6.0 |  | 56.7 ± 10.6 | 52.9 ± 10.4 |  |  |  | 0.042* |  | 0.85* |  |
| iNPH scale  score | 51.5 ± 15.1 | 56.9 ± 15.8 |  | – | – | – | 66.8 ± 17.2 | 60.0 ± 11.3 |  | – | – | 0.018* |  | – | – |
| Scalp to cortex distance (mm)  Hand  Foot | 12.6 ± 1.7  11.5 ± 1.6 | 12.9 ± 1.6  11.6 ± 1.7 |  | 12.4 ± 1.6  11.2 ± 1.8 | 13.4 ± 1.7  12.3 ± 1.4 |  | 13.8 ± 1.3  12.8 ± 1.5 | 13.6 ± 2.4  12.9 ± 1.7 |  |  |  | 0.018* | 0.018* |  |  |
| MT  Hand  %-MSO  EF (V/m)  Foot  %-MSO  EF (V/m) | 29.1 ± 7.0  93.0 ± 25.3  46.7 ± 7.4  185.9 ± 33.1 | 26.6 ± 5.9  83.1 ± 15.6  38.9 ± 6.1  121.4 ± 24.3 | 0.001* | 27.9 ± 5.7  86.3 ± 16.9  48.0 ± 8.8  158.6 ± 38.8 | 30.0 ± 5.5  88.0 ± 12.1  42.6 ± 6.4  130.0 ± 31.2 |  | 31.1 ± 6.8  89.0 ± 21.7  48.9 ± 9.4  137.6 ± 25.5 | 29.6 ± 6.7  82.6 ± 12.8  42.0 ± 6.3  117.7 ± 14.4 |  |  |  | 0.045* | 0.027* | 0.80*  0.86* |  |
| SP  Duration (ms) | 54.7 ± 16.7 | 51.3 ± 14.8 |  | 51.9 ± 16.5 | 60.7 ± 22.6 |  | 53.8 ± 16.7 | 62.2 ± 18.6 |  |  |  |  |  |  |  |
| IO  MAX (μV)  V50 (%-MSO)  Slope | 2686.3 ± 1508.2  39.1 ± 9.3  3.4 ± 1.3 | 6086.4 ± 2665.8  35.8 ± 10.7  2.2 ± 1.4 | 0.012* | 3311.9 ± 1241.9  38.2 ± 7.8  3.7 ± 1.6 | 4796.0 ± 2801.8  40.4 ± 8.2  3.0 ± 1.3 |  | 4841.1 ± 1859.1  42.8 ± 11.2  4.3 ± 2.2 | 5055.7 ± 2416.6  38.3 ± 11.6  2.4 ± 1.7 |  |  |  | 0.018* |  |  |  |
| RS  2nd MEP  3rd MEP  4th MEP  (MEP amplitudes were normalized by the amplitude of the 1^st^ MEP in the RS trials) | 0.80 ± 0.18  0.79 ± 0.21  0.79 ± 0.29 | 0.78 ± 0.20  0.82 ± 0.30  0.79 ± 0.25 |  | 0.75 ± 0.14  0.73 ± 0.19  0.74 ± 0.20 | 0.72 ± 0.24  0.66 ± 0.19  0.69 ± 0.20 |  | 0.79 ± 0.30  0.93 ± 0.43  0.98 ± 0.57 | 0.75 ± 0.22  0.79 ± 0.20  0.74 ± 0.15 |  |  |  |  |  |  | 0.90* |
| Map  Hand  Area  COG  X  Y  Z  Foot  Area  COG  X  Y  Z | 2.3 ± 1.9  125.7 ± 5.1  190.8 ± 4.8  82.7 ± 7.1  2.3 ± 1.8  108.4 ± 5.8  192.7 ± 9.0  68.5 ± 7.6 | 2.4 ± 2.3  127.4 ± 3.8  190.5 ± 6.0  91.6 ± 7.2  3.0 ± 2.2  105.4 ± 4.4  199.2 ± 6.1  81.6 ± 7.9 | 0.047*  0.011* | 1.7 ± 1.1  130.5 ± 3.7  187.6 ± 3.9  83.2 ± 9.4  1.7 ± 0.7  106.1 ± 4.3  193.5 ± 9.9  69.6 ± 10.7 | 2.4 ± 2.1  126.7 ± 3.2  189.9 ± 6.6  91.2 ± 9.2  3.4 ± 1.5  108.0 ± 3.7  198.5 ± 7.3  81.4 ± 8.5 | 0.030* | 2.9 ± 1.5  127.3 ± 5.0  186.3 ± 6.1  83.5 ± 6.3  2.4 ± 1.1  103.6 ± 2.6  192.4 ± 9.0  74.3 ± 4.5 | 2.4 ± 1.2  124.4 ± 3.0  191.2 ± 5.9  92.8 ± 12.2  3.7 ± 1.7  100.8 ± 2.5  200.5 ± 3.3  83.2 ± 10.5 |  | 0.028*  0.028* |  | 0.046* | 0.046* | 0.83* | 1.00* |

**Data of good responders and limited responders at baseline, TAP and follow-up.**

Study population was divided into two subgroups of seven subjects. Cutoff point for subgroups was 17% improvement in walking time from baseline to follow up (good responders >17% and limited responders <17%)

SP = Silent period, RMT = Resting motor threshold, EF = Electric field, IO = Input-Output curve, RS = Repetition Suppression, BBT = Box and Block Test, GPT = Grooved Pegboard Test, iNPH = idiopathic normal pressure hydrocephalus, %-MSO = percentage of maximum stimulator output, EF = Electric field, MAX = maximum value of the curve, V50 = The mid-point of the curve, Slope = the slope of the curve, MEP = motor evoked potential, Map = Mapping of the cortical representation areas, COG = centers-of-gravity (X, Y, Z -coordinates), TAP = Time point after TAP test

^1^Wilcoxon signed-rank test was used to analyze differences between each time points.

^2^Changes from baseline to TAP test and from baseline to follow up were calculated. Spearman’s test was used to analyze correlation.

^3^Subgroups were compared by Mann-Whitney U test in each time point.

* *p* < 0.05
